# Cognitive behavioral therapy’s impact on anxiety, depression, and quality of life in patients with head and neck cancer: a systematic review

**DOI:** 10.3389/fpsyg.2025.1547999

**Published:** 2025-09-09

**Authors:** Xi Wang, Wenjuan Li, Tingting Zhao, Qi Jin, Huan Wang

**Affiliations:** ^1^School of Nursing, Gansu University of Chinese Medicine, Lanzhou, China; ^2^Department of Anesthesiology, Gansu Provincial Hospital of TCM, Lanzhou, China

**Keywords:** head and neck cancer, cognitive behavioral therapy, anxiety, depression, quality of life, systematic review

## Abstract

**Objective:**

Patients with head and neck cancer frequently encounter challenges related to emotional fluctuations and psychological distress. Current research uniformly suggests that cognitive behavioral therapy has considerable potential in clinical settings for alleviating emotional issues and improving the quality of life in cancer patients. However, the therapeutic efficacy of CBT specifically for patients with head and neck cancer remains uncertain. This paper aims to systematically evaluate the intervention effects of cognitive behavioral therapy on anxiety, depression, and quality of life among patients with head and neck cancer.

**Method:**

A comprehensive search was conducted across 11 databases, covering the period from the inception of the databases to May 2024. Four reviewers were responsible for study selection, data extraction, and quality assessment. This systematic review was conducted in accordance with the Preferred Reporting Items for Systematic Reviews and Meta-Analyses (PRISMA) guidelines.

**Results:**

Six randomized controlled trials were included, encompassing a total of 657 patients with head and neck cancer. The systematic review revealed that anxiety scores (SMD = −0.61, 95% CI: −1.02 to −0.20, *p* = 0.003) and depression scores (SMD = −0.83, 95% CI: −1.38 to −0.29, *p* = 0.003) were significantly lower in the CBT group compared to the control group; however, the effect of CBT on improving the quality of life (SMD = 0.56, 95% CI: −0.15 to 1.26, *p* = 0. 122) was not statistically significant.

**Conclusion:**

Cognitive Behavioral Therapy is an effective approach for alleviating anxiety and depression in patients with head and neck cancer; however, its impact on improving their quality of life is not significant. The observed heterogeneity across studies may be attributed to several factors such as intervention program design, sample size, outcome assessment tools, and external social influences. Future research should employ more rigorous methodological designs and incorporate larger-sample, multicenter randomized controlled trials to further validate the effectiveness of CBT.

**Systematic review registration:**

https://www.crd.york.ac.uk/PROSPERO/view/CRD42024583519, identifier CRD42024583519.

## Introduction

Head and Neck Cancer (HNC) is among the most common types of cancer worldwide. According to epidemiological statistics, approximately 650,000 new cases of HNC are diagnosed globally each year, with about 325,000 patients succumbing to the disease (Gormley et al., 2022). Head and neck cancer, as a type of malignancy that significantly impacts patients’ appearance and functional abilities, is prone to causing issues such as speech dysfunction, swallowing disorders, and facial disfigurement during the course of the disease and its treatment. These problems render patients more susceptible to social avoidance, feelings of inferiority, and shame, thereby trapping them in a vicious cycle of isolation and emotional distress. Such conditions exert substantial adverse effects on patients’ physical and psychological well-being, subsequently triggering psychological issues like anxiety and depression, and notably diminishing their quality of life (Parker et al., 2014). Therefore, patients with head and neck cancer often experience a higher degree of psychological distress compared to those with other types of cancer, and their need for psychological care is consequently more urgent. Currently, the primary psychological treatment modalities for head and neck cancer patients include pharmacological and non-pharmacological interventions. Among these, non-pharmacological psychotherapies are widely applied due to their minimal side effects and favorable therapeutic outcomes (Chen et al., 2024). A meta-analysis indicates that non-pharmacological therapies, such as structured psychotherapy, mindfulness-based stress reduction programs, and interventions aimed at enhancing psychological resilience, hold significant clinical value for cancer patients. These approaches not only show promise in optimizing mental health but also improve patients’ quality of life throughout the cancer trajectory, providing clear evidence for their effectiveness in substantially alleviating pain, anxiety, and depression among cancer patients (Paslaru et al., 2025).

Among various approaches, Cognitive Behavioral Therapy (CBT), as a structured and goal-oriented psychological intervention method (Thoma et al., 2015), works by modifying patients’ cognitive and behavioral patterns to break the cycle of negative emotions and enhance their psychological resilience and coping abilities. It aids head and neck cancer patients in adopting a more positive perspective on their own changes, strengthening their connections with others, thereby alleviating internal conflicts and rebuilding a sense of hope and control over their lives. This series of intervention mechanisms not only helps alleviate negative emotions in patients with head and neck cancer but may also indirectly enhance their treatment adherence and overall quality of life. Currently, CBT has been widely utilized in the psychotherapy of cancer patients (Dils et al., 2024).

However, current research predominantly focuses on the application of CBT in cancer patients. According to a meta-analysis evaluating the efficacy of CBT in alleviating anxiety and depression among cancer patients, compared to standard treatment, CBT can effectively reduce anxiety and depression scores in this population (Zhang L. et al., 2022). Additionally, a systematic review and meta-analysis encompassing 154 studies on cancer patients demonstrated that CBT can significantly improve patients’ health status, enhance their cognitive abilities, and elevate their quality of life (Dils et al., 2024). Another systematic review and meta-analysis focusing on evaluating the mental health and quality of life of elderly cancer patients yielded consistent findings (O'Keefe et al., 2024). However, the majority of existing studies have merely conducted a straightforward pooled analysis of the effects of CBT on different cancer patient populations, failing to delve into an in-depth analysis and comparison of specific CBT intervention methods, as well as neglecting to explore the differential intervention effects of CBT across various cancer patient groups.

Currently, there is a lack of systematic summarization regarding the therapeutic efficacy of CBT specifically in the population of head and neck cancer patients. Whether CBT can effectively alleviate anxiety and depression, as well as improve quality of life in this group, remains to be further validated. Additionally, notable discrepancies exist in previous studies concerning patient selection criteria, intervention modalities, and outcome assessment tools, which have constrained our comprehensive understanding of CBT’s efficacy. Therefore, this study will employ a systematic review approach, incorporating all relevant randomized controlled trials (RCTs), to investigate the impact of CBT on anxiety, depression, and quality of life among head and neck cancer patients.

## Methods

### Protocol and registration

This systematic review was conducted in accordance with the Preferred Reporting Items for Systematic Reviews and Meta-Analyses (PRISMA) guidelines (Page et al., 2021). The study is registered in the Prospective Register of Systematic Reviews (PROSPERO) with the registration number CRD42024583519. Supplementary Table S1 presents the PRISMA (Preferred Reporting Items for Systematic Reviews and Meta-Analyses) checklist.

### Data sources and searches

To mitigate publication bias, a comprehensive review was conducted that included both published and unpublished clinical trials. We conducted a comprehensive search of the Cochrane Library, PubMed, Embase, Web of Science, Scopus, PsycINFO, CINAHL, CNKI, CBM, VIP, and Wan Fang Data (CDDB) databases from their inception through May 2024. The database search details are shown in Table 1. The search strategy for this review adhered to the Cochrane guidelines for systematic reviews, using a combination of MeSH terms and free-text words, taking the PubMed database as an example.

**Table 1:** PubMed database search.

Databases: PubMed	
#1	(Head and neck neoplasm*[MeSH Terms] OR Head and neck neoplasm* [Title/Abstract] OR Head and Neck malignanc*[Title/Abstract] OR Head and neck carcinoma*[Title/Abstract] OR Head and neck cancer*[Title/Abstract] OR Head and neck tumor*[Title/Abstract])
#2	(Cognitive behavioral therapy[MeSH Terms] OR CBT[Title/Abstract] OR cognitive-behav*[Title/Abstract] OR cognitive behav*[Title/Abstra ct] OR cognitive therapy[Title/Abstract] OR behavior therapy[Title/Abs tract] OR cognitive behavioral therapy[Title/Abstract] OR psycholog* [Title/Abstract])
#3	#1 AND #2

The following filters were applied in all databases if possible: type of publication (controlled trials only), species (humans), and language (English).

To ensure a thorough identification of relevant studies, both published and unpublished, as well as ongoing trials, we undertook the following steps: (1) examined the reference lists of identified articles and performed citation tracking of early systematic reviews related to psychological interventions, and (2) searched for ongoing trials and study registrations in databases such as ClinicalTrials.gov^1^ and PROSPERO,^2^ continuing until no additional pertinent studies were found (van der Meulen et al., 2014; Liu et al., 2021; Graboyes et al., 2025; Liu et al., 2023; Chopra et al., 2024; Zhao et al., 2024).

### Eligibility criteria

#### Inclusion criteria

According to the PICOS framework, Table 2 provides a detailed illustration of the inclusion criteria established in this systematic review.

1. Participants (P): Patients aged 18 years and older with head and neck cancer, including but not limited to oral cancer, nasopharyngeal cancer, laryngeal cancer, etc.;
2. Intervention (I): CBT as the primary intervention strategy;
3. Comparator (C): Patients receiving standard care (not including CBT intervention);
4. Outcomes (O): anxiety, depression and health-related quality of life;
5. Study Design (S): Randomized controlled trial, only studies that have obtained ethical approval and are published in English or Chinese are included in the analysis.

**Table 2:** Inclusion criteria according to PICOS strategy.

PICO strategy	Inclusion criteria
Participants	Patients 18 years of age and older with malignant tumors of the head and neck, including but not limited to oral cancer, nasopharyngeal cancer, laryngeal cancer, etc.
Intervention	CBT as the primary intervention strategy.
Comparison	Patients receiving standard care (not including CBT intervention).
Outcomes	Anxiety, depression and health-related quality of life.
Study design	Randomized controlled trial (RCTs).

#### Exclusion criteria

1. Studies that failed to employ validated instruments for outcome measurement;
2. Studies lacking a well-defined methodological framework;
3. Duplicate publications or multiple articles reporting identical data (only the most comprehensive article in terms of research information was retained).

The evaluation of all prior studies was conducted independently by two investigators, with any disagreements concerning the inclusion or exclusion of studies being resolved through a consensus-based approach.

### Data management and study selection

We used Endnote software to import and manage the files. Two authors (WX and ZT) independently screened the titles and abstracts of the articles and then reviewed the full texts. Any discrepancies were resolved through discussion with a third author (JQ) until a consensus was reached.

### Risk of bias

The Risk of Bias (RoB) for included studies was assessed using the Cochrane Collaboration’s Risk of Bias assessment tool (Higgins et al., 2011). This tool evaluates the RoB across six domains: selection bias, performance bias, detection bias, attrition bias, reporting bias, and other biases. Studies were categorized as having a high risk of bias (if at least one domain was assessed as high risk), an unclear risk (if at least one domain was assessed as unclear and other domains were low risk), or a low risk of bias (if all individual domains were assessed as low risk).

### Data extraction

Following the removal of duplicates, this review adhered to the Cochrane Handbook for Systematic Reviews. Two authors (WX and WH) independently extracted data by reading the full texts and utilized a standardized data extraction form (Microsoft Excel) to extract information from all eligible studies. A third independent person (JQ) checked the data extraction process. The following information was extracted: author, year, country, sample size, age, cancer stage, type of intervention, duration, follow-up, and outcomes.

### Statistical analysis

All statistical analyses for this study were conducted using Stata 18.0 software.

The raw data included in this study pertained to continuous variables, and a random effects model was utilized for the meta-analysis. The outcomes of this meta-analysis are presented as standardized mean differences (SMD) along with 95% confidence intervals (CI). The standardized mean difference serves as a measure of the effect size, indicative of the impact of CBT on patients with head and neck cancer. Each effect size is accompanied by a 95%CI, with a *p*-value of less than 0.05 denoting statistical significance. The I^2^ statistic and *p*-value were employed to evaluate the heterogeneity among studies. An I^2^ value less than 50% and a *p*-value greater than 0.1 suggested a lack of significant statistical heterogeneity among the study results, warranting the use of a fixed-effects model for the meta-analysis. Conversely, an I^2^ value of 50% or more and a *p*-value less than 0.1 indicated significant statistical heterogeneity, leading to the application of a random-effects model. The origins of heterogeneity were further investigated through subgroup analysis and sensitivity analysis. Statistical significance was accepted at the level of *p* < 0.05.

## Results

### Selection and inclusion of studies

After an electronic literature search, a total of 4,841 references were retrieved.

**Figure 1:**
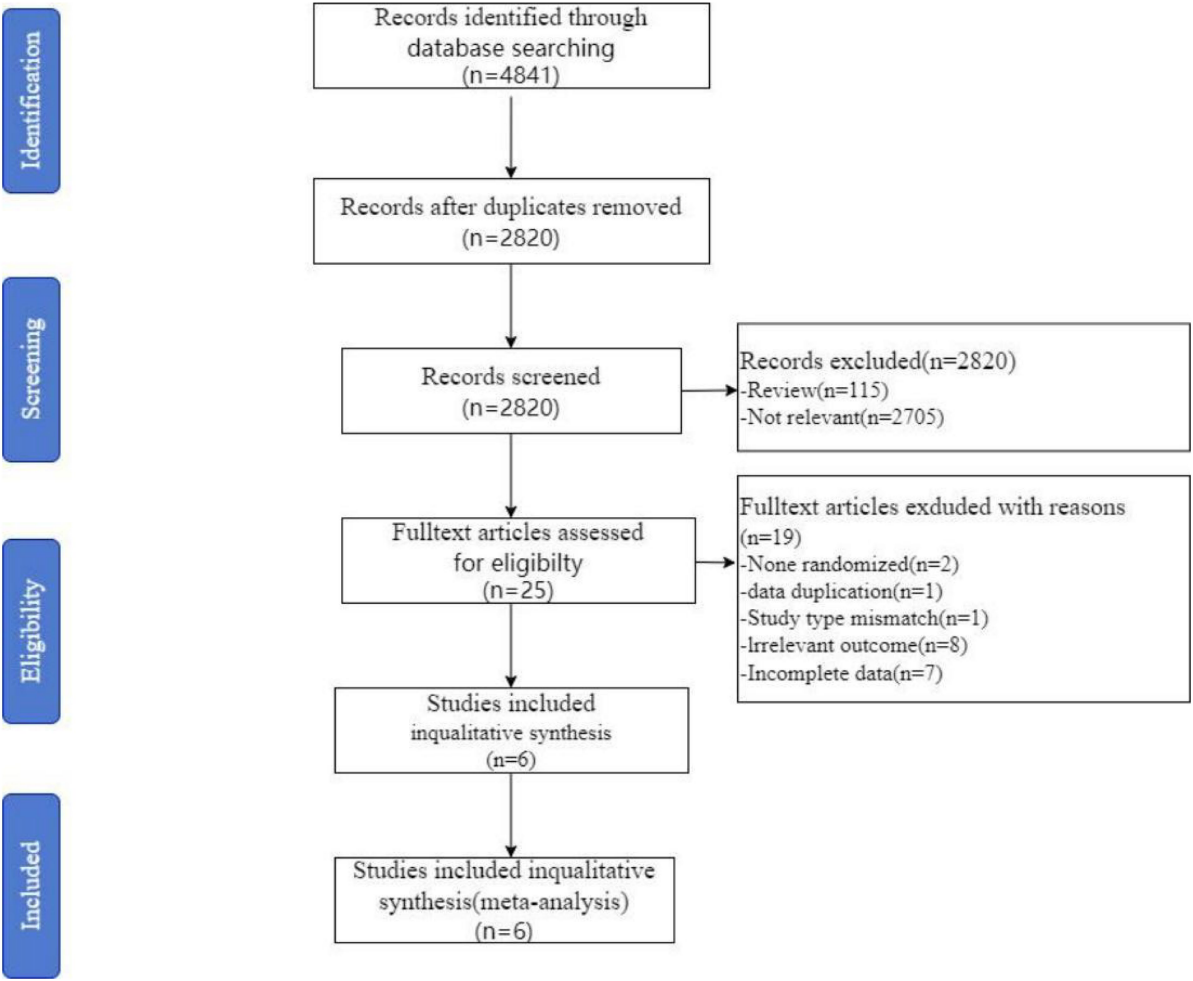
Preferred Reporting Items for Systematic Reviews and Meta-analyses (PRISMA) flowchart from record identification to study inclusion.

The results of the literature search and screening process are summarized in Figure 1. After removing duplicates and screenings, 6 RCTs were included in the study. During the full-text screening phase, a total of 19 articles were excluded, with a detailed list provided in Supplementary Table S2.

### Characteristics of all the included studies

The six studies included in this review were published between 2014 and 2024.

This study encompasses patients with head and neck cancer from three different countries, with three studies (50%) conducted in China, two studies (33.3%) in the United States, and one study (16.7%) in Netherlands. The patient populations assessed included head and neck cancer patients in three studies (50%), nasopharyngeal cancer patients in one study (16.7%), thyroid cancer patients in one study (16.7%), and laryngeal cancer patients in one study (16.7%). All six studies (100%) employed Cognitive Behavioral Therapy (CBT) as the primary intervention, with four studies (66.7%) using conventional nursing care as the control intervention, one study (16.7%) using remote supportive care, and one study (16.7%) using telephone follow-up. Treatment planning was weekly in three studies (50%), once every 2 months in one study (16.7%), a single intervention in one study (16.7%), and one study (16.7%) did not report the frequency and duration of interventions. The treatment format was face-to-face in five studies (83.3%) and remote video intervention in one study (16.7%).

The sample size for the intervention groups (IG) ranged from 20 to 115 cases, and for the control groups (CG) from 19 to 113 cases. The average age range for both IG and CG was 38 to 60 years, with a total of 657 participants involved in this systematic review. Of these, 326 participants were in the IG and 331 in the CG. The attrition rate at the end of the studies ranged from 5 to 25%. The number of treatment sessions varied from one to six, with each session lasting from a minimum of 30 min to a maximum of 60 min, except for one article that did not report the number of interventions (Liu et al., 2023). Three interventions (50%) were primarily delivered by a collaborative team of nurses, psychiatrists, and psychologists, two interventions (33.3%) were conducted solely by clinical psychologists, and one intervention (16.7%) (van der Meulen et al., 2014) was provided only by nurses. Detailed information on the characteristics of these studies can be found in Table 3 of the research.

**Table 3:** Characteristics of the controlled trials included in the systematic review.

No	Author (year)	Country	Sample size		Age (years)	Cancer stage	Intervention: l—Type; 2—Duration; 3—Follow-up	Outcome (scale)
			T	C				
[1]	Liu et al. (2021)	China	115	113	18–70	III/IV	CBT plus; 6 sessions (1 session per week) for 45 min; At baseline, at completion of radiotherapy, and at 6, 12 and 24 months after radiotherapy	HADS
[2]	van der Meulen et al. (2014)	Netherlands	103	102	Mean age: IG: 60.1 CG: 60.7	I–II/III–IV/Unknown	NUCAI; 6 sessions (1 session per 2 months) for 60 min; At baseline, i.e., before starting cancer treatment, and at 3, 6, 9, 12 (i.e., completion of NUCAI), 18, and 24 months after completion of cancer treatment	CES-D; EORTC QLQ-C30 and QLQ H&N35
[3]	Graboyes et al. (2023)	American	20	24	41–80	I-II/III-IV	Psychologist-led tele-CBT sessions (BRIGHT); 5 sessions (1 session per week) for 60 min; At baseline and at 1 and 3 months after intervention	PROMIS SF v1.0—Anxiety; PROMIS SFv1.0—Depression
[4]	Zhao et al. (2024)	China	48	49	Mean age: IG: 38.69 CG: 38.35	I, II, III, IV	CBT-HEP intervention; During the hospital stay, a single intervention session was conducted, which duration ranged from 30 to 60 min; Prior to the intervention, following the intervention, and at the 6-month follow-up post-intervention.	HAMA; PHQ-9; EORTC QLQ-C30
[5]	Liu et al. (2023)	China	35	35	Mean age: IG: 53.21 CG: 52.23	No report	Routine nursing mode plus cognitive behavioral nursing intervention team; During the hospital stay, interventions were conducted, but the specific number of interventions and the frequency of these interventions were not reported; No report.	HAMA; HAMD; QLICP-HN
[6]	Chopra et al. (2024)	American	20	19	Mean age: IG: 58.6 CG: 56.5	I, II, III, IV	4 counseling sessions; 4sessions (1 session per week) for 60 min; At baseline and after completion of the intervention at week 4.	FACIT-H&N

T, treatment group; C, control group; HADS, Hospital Anxiety and Depression Scale; CES-D, Center for Epidemiologic Studies Depression Scale; EORTC QLQ-C30 and QLQ H&N35, European Organisation for Research and Treatment of Cancer Quality of Life Questionnaire Core 30 and European Organisation for Research and Treatment of Cancer Quality of Life; PROMIS SF v1.0-Anxiety, Patient-Reported Outcomes Measurement Information System Short Form v1.0—Anxiety Scale; PROMIS SFv1.0-Depression, Patient-Reported Outcomes Measurement Information System Short Form v1.0—Depression Scale; HAMA, Hamilton Anxiety Rating Scale; PHQ-9, Patient Health Questionnaire-9; HAMD, Hamilton Depression Rating Scale; QLICP-HN, Quality of Life Instrument for Cancer Patients-Head and Neck Module; FACIT-H&N, Functional Assessment of Chronic Illness Therapy—Head and Neck.

### Bias risk assessment

**Figure 2:**
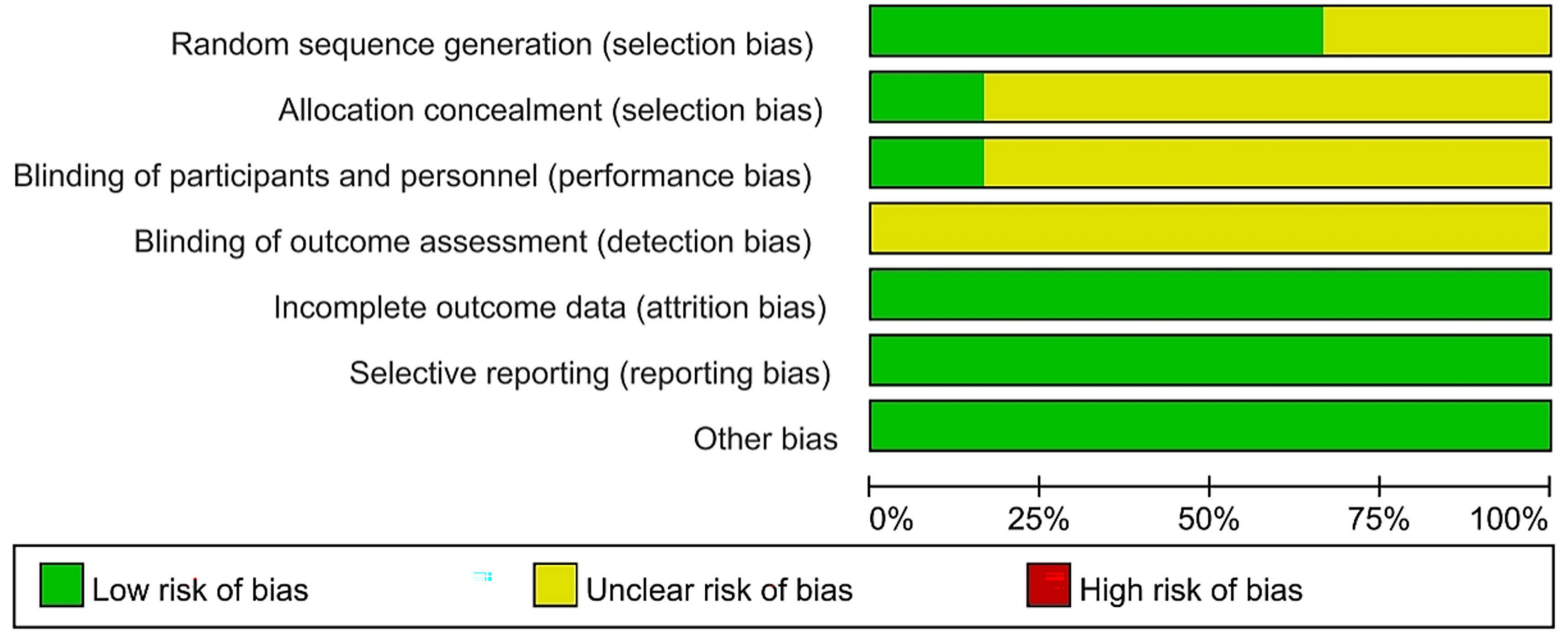
Risk of bias graph of included studies.

**Figure 3:**
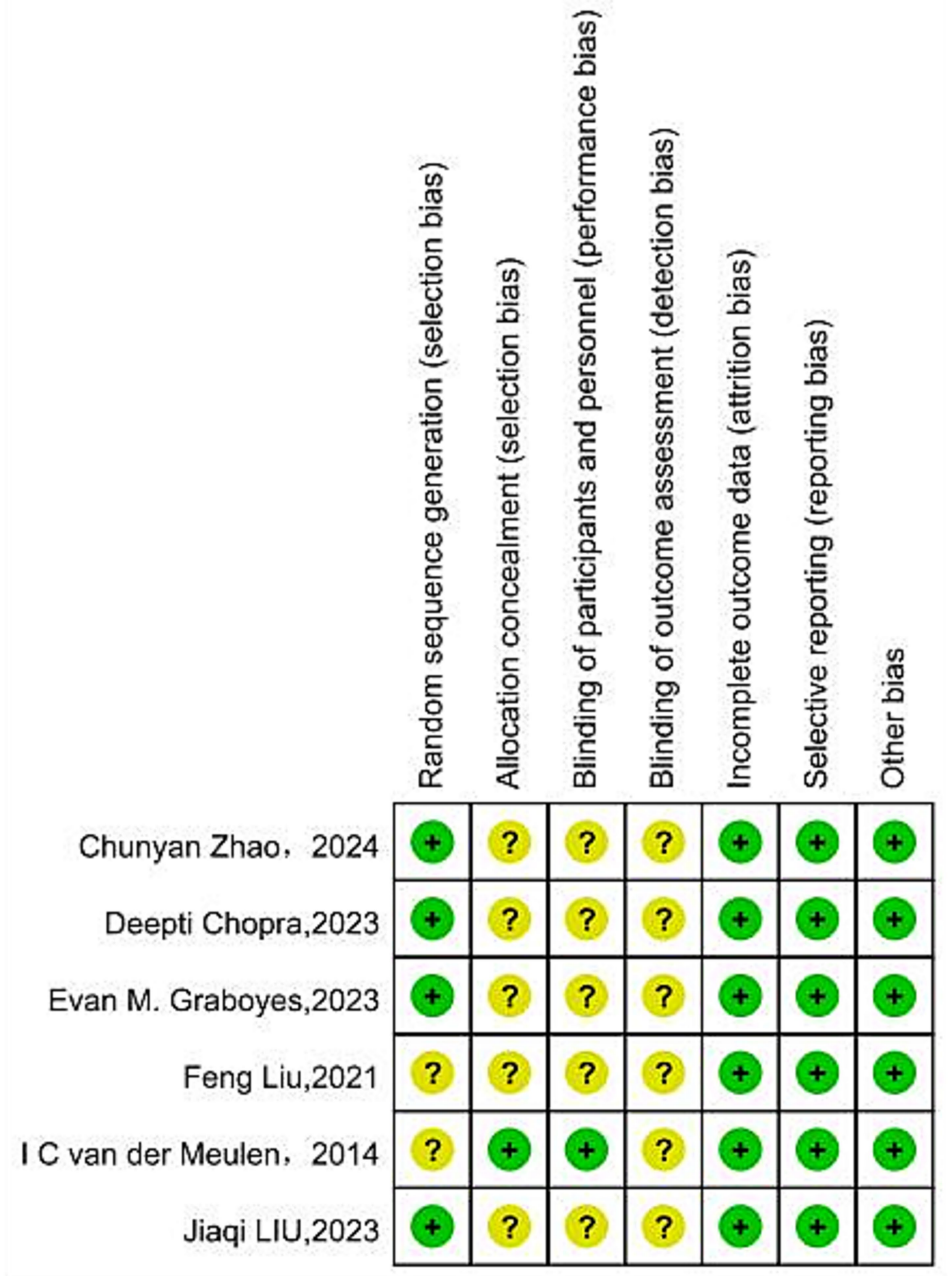
Risk of bias summary of included studies.

Among the included studies, methodological quality varied from low to moderate bias. Two reviewers independently assessed study quality using the Cochrane Collaboration’s Risk of Bias tool. Of the six studies included, One study (van der Meulen et al., 2014) was rated ‘A’ for low bias, while five were graded ‘B’ for moderate bias. All six included RCTs (100%) mentioned the use of randomization for group allocation; four articles (66.7%) described the specific methods and processes for generating the random sequence; one article (16.7%) (van der Meulen et al., 2014) reported the use of concealed allocation; due to the challenges in blinding participants and personnel in psychological interventions, only one RCT (16.7%) (van der Meulen et al., 2014) reported the implementation of blinding for participants and data collectors; two articles (33.3%) (van der Meulen et al., 2014; Liu et al., 2021) reported on patient withdrawal or loss to follow-up, with reasons provided for each. The results of the quality assessment for each study are presented in Figures 2, 3.

### Data synthesis

#### Anxiety

**Figure 4:**
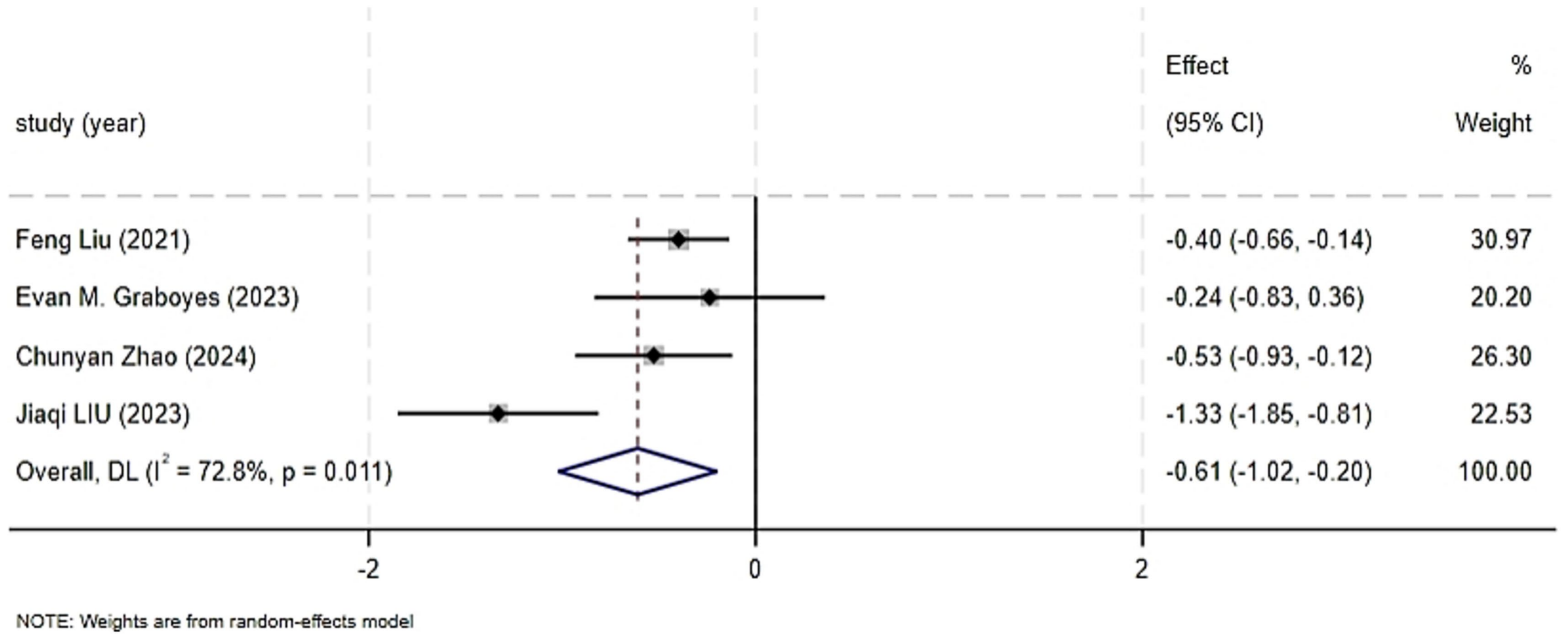
Forest graph showing analysis of Head and neck cancer patient anxiety score of Head and neck cancer patients in CBT group and control group.

The individual results of each study included in this systematic review are presented in Supplementary Table S3. Four randomized controlled trials reported on the impact of CBT on patient anxiety, encompassing a total of 439 patients (Liu et al., 2021; Graboyes et al., 2025; Liu et al., 2023; Zhao et al., 2024). The systematic review revealed a significant improvement in anxiety levels in the intervention group (SMD = −0.61, 95%CI: −1.02 to −0.20, *p* = 0.003). High heterogeneity was observed among the studies (I^2^ = 72.8%, *p* = 0.011), necessitating the use of a random-effects model to account for the variability between the study results. The forest plot for the combined effect estimate of anxiety is presented in Figure 4. Sensitivity analysis was conducted by systematically excluding each study, and when the study with a higher risk of bias (Liu et al., 2023) was excluded, the heterogeneity reduced to zero (I^2^ = 0.0%, *p* = 0.724). Using a fixed-effects model, the results still indicated a statistically significant difference in anxiety scores between the groups (SMD = −0.41, 95%CI: −0.62 to −0.21, *p* = 0.000). As previously mentioned, the exclusion of the Jiaqi LIU study reduced heterogeneity, which may be attributed to differences in study design and content.

#### Depression

**Figure 5:**
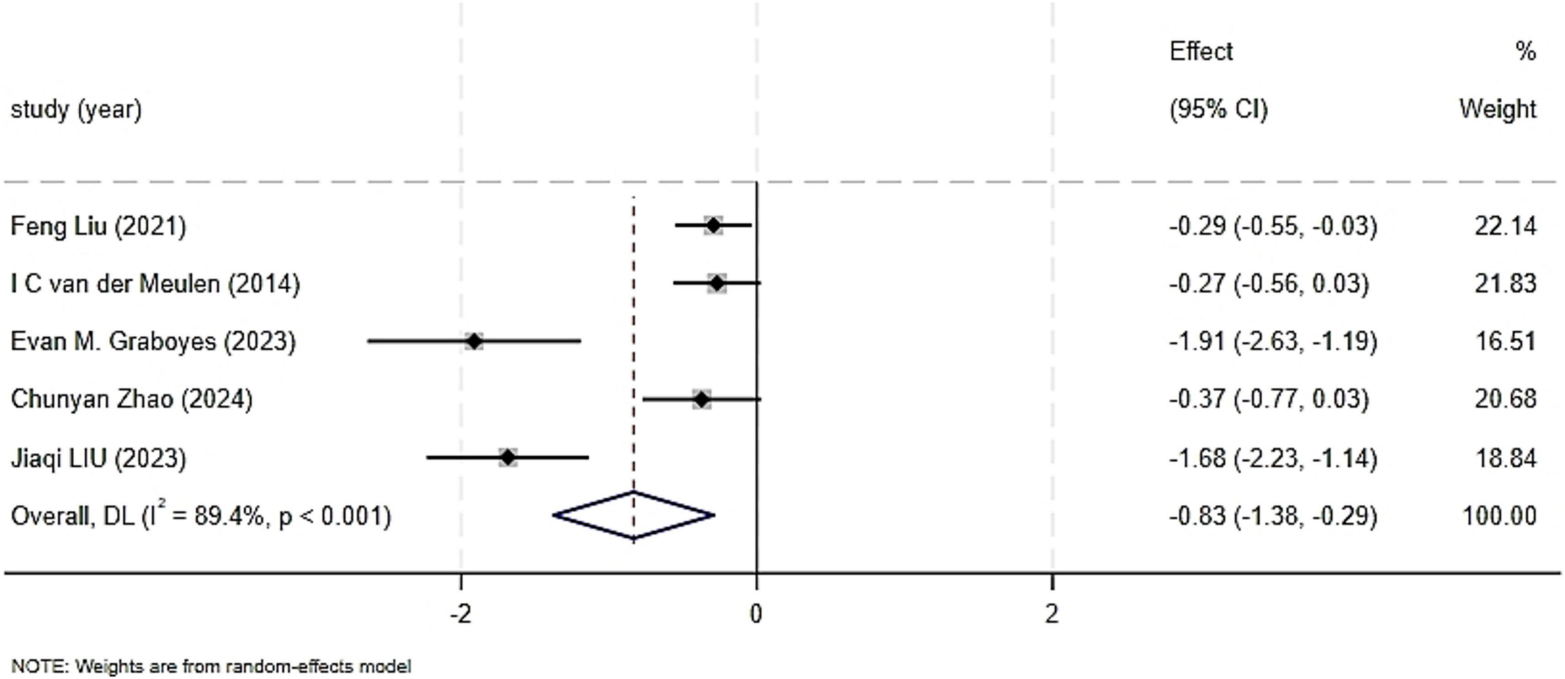
Forest graph showing analysis of Head and neck cancer patient depression score of Head and neck cancer patients in CBT group and control group.

Five trials reported on the effect of CBT on depression, including 618 patients (van der Meulen et al., 2014; Liu et al., 2021; Graboyes et al., 2025; Liu et al., 2023; Zhao et al., 2024). Significant heterogeneity was detected among the included studies (I^2^ = 89.4%, *p* < 0.001), with I^2^ ≥ 50%, leading to the use of a random-effects model. The results showed that the CBT group had a significant improvement in depression compared to the control group (SMD = −0.83, 95%CI: −1.38 to −0.29, *p* = 0.003), with a statistically significant difference. The forest plot for the combined effect estimate of depression is presented in Figure 5. Sensitivity analysis, which involved sequentially excluding studies, did not significantly change the heterogeneity or the outcomes, indicating robustness of the synthesis. The sources of heterogeneity may be related to the specific intervention content and the different assessment scales used in each study.

#### Quality of life

**Figure 6:**
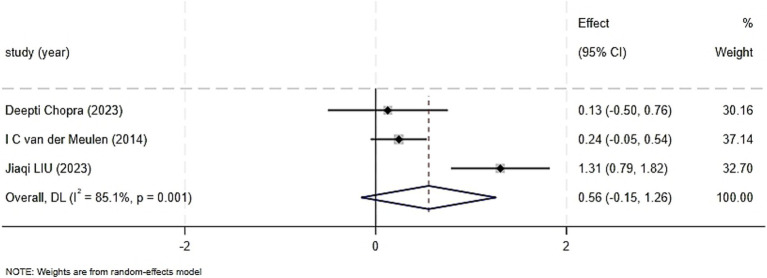
Forest graph showing analysis of Head and neck cancer patient Quality of life score of Head and neck cancer patients in Cognitive behavioral therapy group and control group.

Three trials reported on the impact on quality of life, including 288 patients (van der Meulen et al., 2014; Chopra et al., 2024; Liu et al., 2023). Significant heterogeneity was found among the included studies (I^2^ = 85. 1%, *p* = 0.001), with I^2^ > 50%, prompting the use of a random-effects model. The comparison of the impact of CBT on the quality of life of patients with head and neck cancer showed no statistically significant overall effect (SMD = 0.56, 95% CI: −0.15 to 1.26, *p* = 0. 122). Sensitivity analysis, excluding the study with a higher risk of bias (Liu et al., 2023), resulted in zero heterogeneity among the studies (I^2^ = 0.0%, *p* = 0.745). Using a fixed-effects model, the quality of life scores still did not show a statistically significant difference (SMD = 0.22, 95%CI: −0.05 to 0.49, *p* = 0. 103), indicating robustness of the results, as seen in Figure 6. As discussed earlier, the exclusion of the Jiaqi LIU study led to zero heterogeneity, which may be due to differences in patients’ economic conditions, the medical conditions of the intervention areas, and the individuals implementing the interventions.

#### Upcoming RCT

A randomized controlled trial (RCT) that has been established is aimed at comparing the efficacy of Acceptance and Commitment Therapy (ACT) and Mindfulness-Based Stress Reduction (MBSR) in patients newly diagnosed with head and neck cancer (Zhang Z. et al., 2022). The relevant characteristics and specific details of this study can be found in Supplementary Table S4.

#### Subgroup analysis

**Figure 7:**
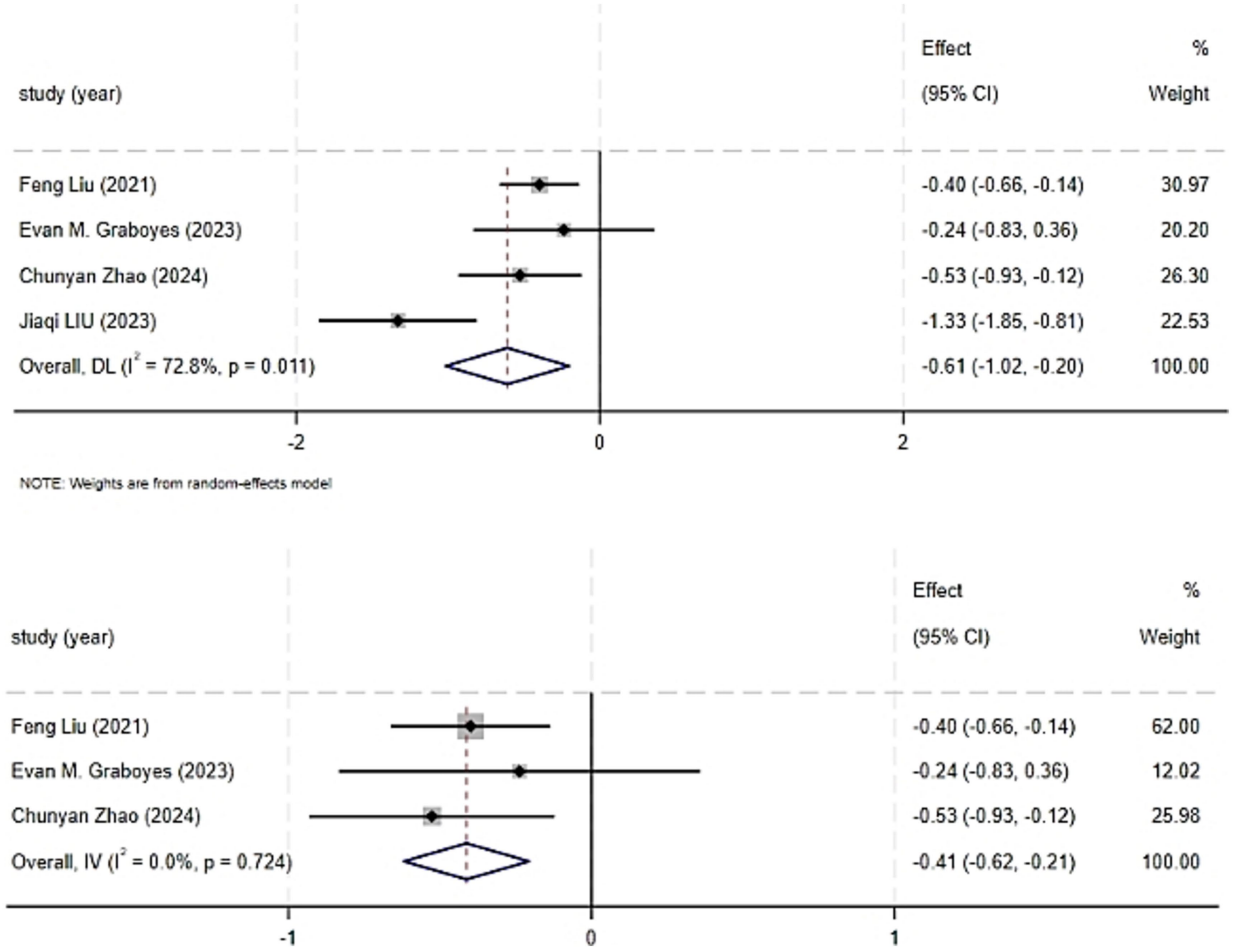
Forest graph showing analysis of anxiety score of Head and neck cancer patients in CBT group and control group.

A subgroup analysis for anxiety was conducted based on the duration of the intervention in four randomized controlled trials, as depicted in Figure 7. When the intervention duration was ≤ 4 weeks, the results were I^2^ = 82.6%, *p* = 0.017; for interventions lasting > 4 weeks, the results were I^2^ = 0.0%, *p* = 0.627. The heterogeneity test revealed a high degree of heterogeneity among the studies (I^2^ = 72.8%, *p* = 0.011).

#### Publication bias

Due to the limited number of articles included in the systematic review (only six studies), a funnel plot analysis was deemed unnecessary.

## Discussion

This study employed a systematic review approach to, for the first time, investigate the intervention effects of CBT on anxiety, depression, and quality of life in patients with head and neck cancer. The results indicated that CBT significantly reduced anxiety and depression scores among these patients, although the improvement in quality of life did not reach statistical significance. This finding aligns with the results of recent systematic reviews (Lin et al., 2024), suggesting that CBT exerts certain positive impacts on psychological outcomes in cancer patients. Such effects may stem from its multi-faceted intervention mechanisms targeting negative emotions, including structured cognitive restructuring, emotion regulation, and behavioral activation strategies. However, the findings of this study diverge from previous research concerning the improvement of quality of life in cancer patients. Specifically, a systematic review and meta-analysis focusing on cancer patients demonstrated that CBT exerts a significant impact on fatigue scores, thereby suggesting its potential positive influence on patients’ overall quality of life (Hosseini Koukamari et al., 2024). The discrepancies mentioned above may be attributed to several factors. Firstly, the limited number of studies and insufficient sample size included in this research—with only three pieces of literature assessing quality of life—resulted in inadequate statistical power. Secondly, varying measurement tools were employed across different studies, such as the EORTC QLQ-C30 and QLICP-HN, which differ in their dimensional structures and sensitivities, thereby affecting the comparability and stability of the research findings. Moreover, improvements in quality of life typically necessitate longer-term interventions and extended follow-up observations to fully manifest. However, the studies incorporated in this systematic review were constrained by limitations in intervention duration and follow-up periods, potentially failing to provide sufficient time for CBT to exert its potential benefits on enhancing quality of life. Lastly, given the multifactorial nature of quality of life, it is influenced not only by the disease itself and psychological state but also by external variables such as the patient’s social support system, economic burden, and family caregiving resources.

The studies included in this systematic review demonstrated substantial heterogeneity in their results, which may be attributed to the following factors. The subgroup analysis of this study revealed that interventions with a duration exceeding 4 weeks exhibited a more stable effect on alleviating anxiety; however, this finding should be interpreted with caution as it is based on only two studies. Furthermore, there remains a notable lack of long-term observation and evaluation regarding the sustained effects of CBT interventions. Future research should focus on clarifying the differences in efficacy between short-term and medium- to long-term interventions, as well as exploring the mechanisms underlying their sustained effects (Cuijpers et al., 2013). Secondly, the six randomized controlled trials (RCTs) included in this study encompassed various types of head and neck cancers; however, most studies did not provide a more detailed classification of head and neck cancer patients. Patients with different cancer types exhibit variations in disease characteristics, treatment modalities, and psychosocial stressors, which may influence the effectiveness of interventions. For instance, laryngeal cancer patients may encounter social barriers due to aphasia or voice impairment, while nasopharyngeal carcinoma patients may experience feelings of shame and loneliness resulting from facial disfigurement. Given the unique clinical and psychosocial needs associated with different cancer types, discrepancies in intervention content may contribute to inconsistent outcomes. Therefore, it is essential to tailor CBT interventions precisely to meet individualized needs.

In addition, the mode of intervention may also impact the efficacy of CBT. A systematic review and meta-analysis have demonstrated that there is little difference in effectiveness between face-to-face CBT and therapist-guided remote CBT across a variety of mental health and somatic conditions (Zandieh et al., 2024). However, its applicability in head and neck cancer patients remains uncertain. Future research should conduct trials to compare the efficacy of different intervention formats in this specific population. Meanwhile, the effectiveness of interventions is constrained not only by their content but may also be influenced by the identity of the intervention providers. A randomized controlled trial revealed that nurse-led cognitive-behavioral interventions could improve the quality of life, self-esteem, and emotional well-being of heart failure patients in the Philippines (Cajanding, 2016). Another systematic review and meta-analysis demonstrated that both psychologists and nurses are effective in delivering group cognitive-behavioral therapy for treating depression without comorbidities, with moderate certainty of evidence (Wong et al., 2024). This is noteworthy because nurses/psychiatric nurses may be more accessible and cost-effective than psychologists in certain contexts. However, for patients with other psychiatric or medical comorbidities, further research is still needed to determine the effectiveness of GCBT delivered by professionals from different backgrounds.

Finally, as most of the studies included in this research did not implement allocation concealment, blinding of assessors, or blinding of investigators in their designs, this adds an extra risk of misleading results and undermines the reliability of the findings. Therefore, future research should, based on randomized controlled trial designs, strive to incorporate allocation concealment, investigator blinding, and assessor blinding to enhance methodological quality. Additionally, a clearer classification of head and neck cancer patients is needed, along with the development of more refined CBT interventions tailored to different types of head and neck cancers.

Based on the results of this study and other related findings, it is recommended that hospitals widely promote CBT and integrate it into the nursing training curriculum. Through systematic training in CBT theory and techniques, more nurses can master the core skills and intervention methods of this therapy, thereby reducing the time cost for patients to receive psychological interventions and enhancing the continuity and stability of the interventions. Secondly, having nurses implement CBT allows for the efficient utilization of existing medical resources without the need to introduce additional professional psychotherapists, simplifying the implementation process and facilitating its integration into routine clinical care. Lastly, as the primary providers of daily patient care, nurses are better positioned to promptly identify patients’ psychological issues and provide immediate interventions, increasing patients’ access to psychological support. This is particularly beneficial in areas or medical settings lacking professional psychotherapists, significantly enhancing the accessibility of CBT services.

It’s worth mentioning that, in addition to CBT, various other psychological intervention methods, such as Mindfulness-Based Stress Reduction (MBSR), expressive writing, and psychoeducation, have been employed in recent years for emotional regulation and quality-of-life improvement in cancer patients. Among these, MBSR utilizes techniques like meditation, mindful breathing, and body scans to enhance patients’ ability to accept their present feelings. It has demonstrated promising results in breast cancer and lung cancer patients (Schell et al., 2019; Aminnasab et al., 2022). Compared to CBT, MBSR places greater emphasis on “non-judgmental awareness,” making it suitable for individuals who struggle to modify negative cognitions. In contrast, CBT focuses on addressing negative thought patterns. For patients with head and neck cancer, CBT and MBSR can be viewed as complementary approaches. In clinical practice, the choice of intervention can be tailored to the individual patient’s characteristics and their primary psychological distress, or a combined intervention model can be explored. For instance, for patients experiencing high levels of anxiety coupled with significant body image concerns, integrating cognitive restructuring from CBT with mindfulness training from MBSR could enhance their self-acceptance and emotional stability. Additionally, some studies have preliminarily validated the feasibility of multi-module intervention models (Hanssen et al., 2019; Gkintoni et al., 2025). In the future, further exploration can be made into integrating CBT with other psychological interventions to meet the diverse psychological support needs of head and neck cancer patients. Through these measures, CBT can more effectively assist patients in improving their psychological state, thereby providing more precise and effective interventions for negative emotions in head and neck cancer patients, helping them better cope with the challenges of the disease, and enhancing their overall quality of life.

### Future research

As more research emerges, it becomes increasingly important to further explore this field, with a particular focus on identifying the key elements within cognitive behavioral therapy that are crucial for its efficacy. To deepen the understanding of the topics discussed in this study, future research should delve into which components are indeed effective, for which patient populations they are effective, and the extent to which universal factors common to all therapies contribute to outcomes. Moreover, these interventions should aim to employ rigorous randomized designs and compare the effects of psychotherapy with other evidence-based treatments.

## Conclusion

CBT has demonstrated positive effects in alleviating anxiety and depression symptoms among head and neck cancer patients; however, it has not shown a significant impact on improving their overall quality of life. This discrepancy may be attributed to factors such as intervention design, sample size, outcome assessment tools, and external social influences. Meanwhile, due to the failure to clarify the impact of these factors on the effectiveness of intervention outcomes, future research should adopt more rigorous methodological designs. This includes strict randomization grouping, reasonable sample size estimation, clear inclusion and exclusion criteria, standardized implementation of blinding methods, uniform intervention protocols, and objective and consistent assessment of outcome indicators, among other aspects. These measures aim to minimize bias to the greatest extent possible, ensuring the reliability and authenticity of research findings, and thereby elucidating the clinical efficacy of CBT in improving anxiety, depression, and quality of life in head and neck cancer patients. Additionally, it is recommended that the psychological state of head and neck cancer patients be incorporated as a routine observation item in nursing care, and that CBT be included in nurse training curricula to enable more nurses to master these scientific psychological intervention methods, thereby helping patients improve their psychological well-being.

## Data Availability

The original contributions presented in the study are included in the article/Supplementary material, further inquiries can be directed to the corresponding author.

## Footnotes

^1^https://www.clinicaltrials.gov/; accessed May 2024.

^2^ https://www.crd.york.ac.uk/PROSPERO/
