# Supplementary material for: Cognitive behavioral therapy’s impact on anxiety, depression, and quality of life in patients with head and neck cancer: a systematic review
Source: Front Psychol. 2025 Sep 9;16:1547999. doi: 10.3389/fpsyg.2025.1547999 (PMC12454428; doi:10.3389/fpsyg.2025.1547999)
Supplement: Supplementary file 1 [file Table_1.DOCX]

Supplementary Material

**Cognitive Behavioral Therapy's Impact on Anxiety, Depression, and Quality of Life in Patients with Head and Neck Cancer: A Systematic Review and Meta-Analysis**

**Xi Wang,Wenjuan Li*,TingTing Zhao,Qi Jin,Huan Wang**

***Correspondence:**

## Wenjuan Li

[13919836610@163.com](mailto:13919836610@163.com)

# Supplementary Table 1

## Protocol adaptations

Since the publication of this scheme, several modifications have been made.The revised title and review question are more clear and precise, aiding readers in quickly comprehending the primary focus and objectives of the study. In our systematic review, we refined the inclusion criteria by incorporating language restrictions for specific study types, ensuring a more targeted and comprehensive assessment of the literature. Furthermore, we elaborated on the statistical methods employed, providing a more detailed description of the analytical procedures beyond the initial framework. This enhanced our ability to interpret the research findings and address potential biases or variations in the data.Due to the heterogeneous characteristics of the clinical trials included in the systematic review and the limited amount of data for each outcome, it was not feasible to conduct the planned meta-analyses and meta-regressions as outlined in the protocol. Consequently, a narrative synthesis was performed in the systematic review.

## PRISMA checklist

| **Section/topic** | **#** | **Checklist item** | **Reported?** |
| --- | --- | --- | --- |
| **TITLE PAGE** | | |  |
| Title | 1 | Identify the report as a systematic review, meta-analysis, or both. | Yes |
| Funding | 2 | Describe sources of funding for the systematic review and other support (e.g., supply of data); role of funders for the systematic review on your title page. | Yes |
| Bulleted statements | 3 | 'Database?' and ' what does this review add?'. | - |
| **ABSTRACT** | | |  |
| Structured summary | 4 | Provide a structured summary including, as applicable: background and objective; databases and data treatment; results, conclusion; systematic review registration number. | Yes |
| **INTRODUCTION** | | |  |
| Rationale | 5 | Describe the rationale for the review in the context of what is already known. | Yes |
| Objectives | 6 | Provide an explicit statement of questions being addressed with reference to participants, interventions, comparisons, outcomes, and study design (PICOS). | Yes |
| **METHODS** | | |  |
| Protocol and registration | 7 | Indicate if a review protocol exists, if and where it can be accessed (e.g., Web address), and, if available, provide registration information including registration number. | Yes |
| Eligibility criteria | 8 | Specify study characteristics (e.g., PICOS, length of follow-up) and report characteristics (e.g., years considered, language, publication status) used as criteria for eligibility, giving rationale. | Yes |
| Information sources | 9 | Describe all information sources (e.g., databases with dates of coverage, contact with study authors to identify additional studies) in the search and date last searched. | Yes |
| Search | 10 | Present full electronic search strategy for at least one database, including any limits used, such that it could be repeated. | Yes |
| Study selection | 11 | State the process for selecting studies (i.e., screening, eligibility, included in systematic review, and, if applicable, included in the meta-analysis). | Yes |
| Data collection process | 12 | Describe method of data extraction from reports (e.g., piloted forms, independently, in duplicate) and any processes for obtaining and confirming data from investigators. | Yes |
| Data items | 13 | List and define all variables for which data were sought (e.g., PICOS, funding sources) and any assumptions and simplifications made. | Yes |
| Risk of bias in individual studies | 14 | Describe methods used for assessing risk of bias of individual studies (including specification of whether this was done at the study or outcome level), and how this information is to be used in any data synthesis. | Yes |
| **Section/topic** | **#** | **Checklist item** | **Reported?** |
| Summary measures | 15 | State the principal summary measures (e.g., risk ratio, difference in means). | Yes |
| Synthesis of results | 16 | Describe the methods of handling data and combining results of studies, if done, including measures of consistency (e.g., I^2^) for each meta-analysis. | Yes |
| Risk of bias across studies | 17 | Specify any assessment of risk of bias that may affect the cumulative evidence (e.g., publication bias, selective reporting within studies). | Yes |
| Additional analyses | 18 | Describe methods of additional analyses (e.g., sensitivity or subgroup analyses, meta-regression), if done, indicating which were pre-specified. | Yes |
| **RESULTS** | | |  |
| Study selection | 19 | Give numbers of studies screened, assessed for eligibility, and included in the review, with reasons for exclusions at each stage, ideally with a flow diagram. | Yes |
| Study characteristics | 20 | For each study, present characteristics for which data were extracted (e.g., study size, PICOS, follow-up period) and provide the citations. | Yes |
| Risk of bias within studies | 22 | Present data on risk of bias of each study and, if available, any outcome level assessment (see item 12). | Yes |
| Results of individual studies | 23 | For all outcomes considered (benefits or harms), present, for each study: (a) simple summary data for each intervention group (b) effect estimates and confidence intervals, ideally with a forest plot. | Yes |
| Synthesis of results | 24 | Present results of each meta-analysis done, including confidence intervals and measures of consistency. | Yes |
| Risk of bias across studies | 25 | Present results of any assessment of risk of bias across studies (see Item 15). | Yes |
| Additional analysis | 26 | Give results of additional analyses, if done (e.g., sensitivity or subgroup analyses, meta-regression [see Item 16]). | Yes |
| **DISCUSSION** | | |  |
| Summary of evidence | 27 | Summarize the main findings including the strength of evidence for each main outcome; consider their relevance to key groups (e.g., healthcare providers, users, and policy makers). | Yes |
| Limitations | 28 | Discuss limitations at study and outcome level (e.g., risk of bias), and at review-level (e.g., incomplete retrieval of identified research, reporting bias). | Yes |
| Conclusions | 29 | Provide a general interpretation of the results in the context of other evidence, and implications for future research. | Yes |

From Moher, D., Liberati, A., Tetzlaff, J., Altman, D. G., & The PRISMA Group. (2009). Preferred Reporting Items for Systematic Reviews and Meta-Analyses: The PRISMA Statement. *PLoS Medicine, 6*(6), e1000097. <https://doi.org/10.1371/journal.pmed1000097>

# Supplementary Table 2

**Characteristics of excluded studies**

| Author (year) | Reason for exclusion |
| --- | --- |
| (Graboyes et al., 2020) | None randomized |
| (Semple et al., 2009) | None randomized |
| (van der Meulen et al., 2013) | data duplication |
| (Kangas et al., 2013) | Study type mismatch |
| (He et al., 2023) | 1rrelevant outcome |
| (ChiCtr, 2019) | 1rrelevant outcome |
| (Graboyes et al., 2023) | 1rrelevant outcome |
| (Graboyes et al., 2022) | 1rrelevant outcome |
| (Patterson, 2019) | 1rrelevant outcome |
| (Maurer et al., 2024) | 1rrelevant outcome |
| (Graboyes et al., 2024) | 1rrelevant outcome |
| (Martino et al., 2022) | 1rrelevant outcome |
| (Richardson et al., 2017) | Incomplete data |
| (Britton et al., 2010) | Incomplete data |
| (Humphris and Ozakinci, 2008) | Incomplete data |
| (Duffy et al., 2006) | Incomplete data |
| (Britton et al., 2017) | Incomplete data |
| (Vaitaitis and Pou, 2019) | Incomplete data |
| (Thilges et al., 2023) | Incomplete data |

**References**

Graboyes, E., Maurer, S., Park, Y., Hill, E., Marsh, C., McElligott, J., et al. (2020). Association of a novel telemedicinebased cognitive behavioral therapy intervention with improved body image disturbance in head and neck cancer survivors: A pilot study. *Psycho-Oncology* 29**,** 30. doi: 10.1002/pon.5327.

Semple, C.J., Dunwoody, L., Kernohan, W.G., McCaughan, E., Semple, C.J., Dunwoody, L., et al. (2009). Development and evaluation of a problem-focused psychosocial intervention for patients with head and neck cancer. *Supportive Care in Cancer* 17(4)**,** 379-388. doi: 10.1007/s00520-008-0480-7.

van der Meulen, I.C., May, A.M., Ros, W.J., Oosterom, M., Hordijk, G.J., Koole, R., et al. (2013). One-year effect of a nurse-led psychosocial intervention on depressive symptoms in patients with head and neck cancer: a randomized controlled trial. *Oncologist* 18(3)**,** 336‐344. doi: 10.1634/theoncologist.2012-0299.

Kangas, M., Milross, C., Taylor, A., and Bryant, R.A. (2013). A pilot randomized controlled trial of a brief early intervention for reducing posttraumatic stress disorder, anxiety and depressive symptoms in newly diagnosed head and neck cancer patients. *PSYCHO-ONCOLOGY* 22(7)**,** 1665-1673. doi: 10.1002/pon.3208.

He, L.L., Xiao, S., Jiang, C.H., Wu, X.W., Liu, W., Fan, C.G., et al. (2023). A randomized, controlled trial to investigate cognitive behavioral therapy in prevention and treatment of acute oral mucositis in patients with locoregional advanced nasopharyngeal carcinoma undergoing chemoradiotherapy. *FRONTIERS IN ONCOLOGY* 13. doi: 10.3389/fonc.2023.1143401.

ChiCtr (2019). Effects of cognitive behavior intervention on nutritional status of patients with head and neck cancer undergoing radiotherapy. *<http://www.who.int/trialsearch/Trial2.aspx?TrialID=ChiCTR1900024910>*.

Graboyes, E.M., Maurer, S., Balliet, W., Li, H., Williams, A.M., Osazuwa-Peters, N., et al. (2023). Efficacy of a Brief Tele-Cognitive Behavioral Treatment vs Attention Control for Head and Neck Cancer Survivors With Body Image Distress A Pilot Randomized Clinical Trial. *JAMA OTOLARYNGOLOGY-HEAD & NECK SURGERY* 149(1)**,** 54-62. doi: 10.1001/jamaoto.2022.3700.

Graboyes, E.M., Maurer, S.N., Balliet, W., Li, H., Williams, A.M., Osazuwa-Peters, N., et al. (2022). Efficacy of a brief telemedicine-based cognitive behavioral treatment (BRIGHT) versus attention control for head and neck cancer survivors with body image distress: A randomized trial. *JOURNAL OF CLINICAL ONCOLOGY* 40(28)**,** 212-212.

Patterson, J.M. (2019). Psychological Interventions for the Head and Neck Cancer Population Who Are Experiencing Dysphagia. *Perspectives of the ASHA Special Interest Groups* 4(5)**,** 1049-1054. doi: 10.1044/2019_PERS-SIG13-2019-0001.

Maurer, S., Balliet, W., Li, H., Williams, A., Osazuwa-Peters, N., Yan, F., et al. (2024). Efficacy of a Brief Tele-Cognitive Behavioral Treatment Versus Attention Control for Head and Neck Cancer Survivors With Body Image Distress: A Randomized Clinical Trial. *PSYCHO-ONCOLOGY* 33.

Graboyes, E.M., Kistner-Griffin, E., Hill, E.G., Maurer, S., Balliet, W., Williams, A.M., et al. (2024). Mechanism underlying a brief cognitive behavioral treatment for head and neck cancer survivors with body image distress. *SUPPORTIVE CARE IN CANCER* 32(1). doi: 10.1007/s00520-023-08248-7.

Martino, R., Khan, M.M., Manduchi, B., Rodriguez, V., Fitch, M.I., Barbon, C.E.A., et al. (2022). EXPLORING PATIENT EXPERIENCES WITH A TELEHEALTH BEHAVIOURAL SWALLOWING INTERVENTION FOR THE PRO-ACTIVE TRIAL IN PATIENTS WITH HEAD AND NECK CANCER. *Supportive care in cancer* 30**,** S177. doi: 10.1007/s00520-022-07099-y.

Richardson, A.E., Tennant, G., Morton, R.P., and Broadbent, E. (2017). A Self-Regulatory Intervention for Patients with Head and Neck Cancer: Pilot Randomized Trial. *ANNALS OF BEHAVIORAL MEDICINE* 51(5)**,** 629-641. doi: 10.1007/s12160-017-9885-1.

Britton, B., Clover, K., Baker, A., and Carter, G.L. (2010). Heads up: A phase II trial of a psychological intervention to reduce malnutrition and depression in head and neck cancer patients undergoing radiotherapy. *Asia-Pacific Journal of Clinical Oncology* 6**,** 191. doi: 10.1111/j.1743-7563.2010.01349.x.

Humphris, G., and Ozakinci, G. (2008). The AFTER intervention: a structured psychological approach to reduce fears of recurrence in patients with head and neck cancer. *Br J Health Psychol* 13(Pt 2)**,** 223-230. doi: 10.1348/135910708x283751.

Duffy, S.A., Ronis, D.L., Valenstein, M., Lambert, M.T., Fowler, K.E., Gregory, L., et al. (2006). A tailored smoking, alcohol, and depression intervention for head and neck cancer patients. *Cancer epidemiology, biomarkers & prevention* 15(11)**,** 2203‐2208. doi: 10.1158/1055-9965.EPI-05-0880.

Britton, B., Baker, A., Clover, K., McElduff, P., Wratten, C., and Carter, G. (2017). Heads Up: a pilot trial of a psychological intervention to improve nutrition in head and neck cancer patients undergoing radiotherapy. *EUROPEAN JOURNAL OF CANCER CARE* 26(4). doi: 10.1111/ecc.12502.

Vaitaitis, V.J., and Pou, A.M. (2019). In-office psychological counseling for head and neck cancer patients. *Otolaryngology - Head and Neck Surgery* 161(2)**,** P74-P75. doi: 10.1177/0194599819858141.

Thilges, S., Mumby, P., Sinacore, J., Clark, J., and Czerlanis, C. (2023). Implementing a cognitive behavioral intervention for patients with head and neck cancer. *SUPPORTIVE CARE IN CANCER* 31(8). doi: 10.1007/s00520-023-07948-4.

# Supplementary Table 3

**TABLE 3** Results of the controlled trials included in this systematic review.

| **Author (year)** | **Results** |
| --- | --- |
| [1]Liu et al.( 2021) | CBT group had significantly lower anxiety and depression scores than TAU group at the end of the intervention, 6 months, 12 months, and 24 months of follow-up (P < .05). |
| [2]van der Meulen et al.(2014) | Intervention group had significantly lower depressive symptoms than control group at the end of the intervention and 6 months of follow-up (P < 0.001). Although the depressive symptoms of the intervention group were not significantly lower than those of the control group at 12 months of follow-up, they were still lower than those of the control group. |
| [3]Graboyes et al. (2023) | The BRIGHT group did not show a significant decrease in anxiety levels at the end of the intervention and 3 months of follow-up, but there was a clear downward trend. In contrast, the AC group's anxiety levels did not show a significant change.At the end of the intervention（P = 0.064）and 3 months of follow-up(P = 0.046), the BRIGHT group had significantly lower depression levels than the AC group . |
| [4]Chunyan et al.(2024) | The CBT-HEP group had significantly lower anxiety levels than the CC group at the end of the intervention (P < 0.001) and 6 months of follow-up (P = 0.002).The CBT-HEP group also had significantly lower depression levels than the CC group at the end of the intervention (P = 0.003) and 6 months of follow-up (P = 0.011). |

**TABLE 3 (Continued)**

| **Author (year)** | **Results** |
| --- | --- |
| [5]Chopra et al.(2023) | The intervention group showed a decrease in anxiety and depression levels after the intervention, but the degree of decrease was not significantly different from that of the control group (P > 0.05). After the intervention, the intervention group had an improvement in overall quality of life and head and neck cancer-specific quality of life, but the degree of improvement was not statistically significant compared with the control group. |
| [6]Jiaqi LIU et al.(2023) | The intervention group had significantly lower anxiety and depression scores than the control group at the end of the intervention (P < 0.05). In addition, the intervention group had significantly higher quality of life scores than the control group at the end of the intervention (P < 0.05). |

# Supplementary Table 4

Upcoming controlled trial

| **Author (year), location, design** | **Target condition** | **Treatment arms (*n*); delivery period (format)** | **Sessions, *n* (minutes)** | **Therapists** | **Assessments**  **(time horizon)** | **Primary outcome**  **(instrument)** | **Secondary outcomes (instruments)** |
| --- | --- | --- | --- | --- | --- | --- | --- |
| Zheng Zhang et al.  (2022),Malaysia  RCT | HNC | ACT,MBSR,  and TAU | Eight sessions  ACT and MBSR(60min); MBSR (150min)  Face-to-face group | Postgraduate students | Pre ;  Post ;  24 weeks post | - Posttraumatic growth   (PTGI-SF) | - Quality of life (FACT-H & N) - Hope (Dispositional Hope Scale) - Optimism (LOT-R) - Experiential avoidance (AAQ-II) - Depression and anxiety (HADS) |

RCT = randomized controlled trial;HNC=head and neck cancer;ACT = acceptance and commitment therapy;MBSR= mindfulness-based stress reduction therapy;TAU = treatment-as-usual;PTGI-SF=**Posttraumatic Growth Inventory-Short Form**;FACT-H & N=Functional Assessment of Cancer Therapy–Head & Neck;LOT-R:Life Orientation Test-Revised;AAQ-II:Acceptance and Action Questionnaire version II ;HADS=Hospital Anxiety and Depression Scale.
